# Full Factorial Analysis of Mammalian and Avian Influenza Polymerase Subunits Suggests a Role of an Efficient Polymerase for Virus Adaptation

**DOI:** 10.1371/journal.pone.0005658

**Published:** 2009-05-21

**Authors:** Olive T. W. Li, Michael C. W. Chan, Cynthia S. W. Leung, Renee W. Y. Chan, Yi Guan, John M. Nicholls, Leo L. M. Poon

**Affiliations:** 1 State Key Laboratory of Emerging Infectious Diseases, Department of Microbiology, The University of Hong Kong, Hong Kong SAR, China; 2 Department of Pathology, The University of Hong Kong, Hong Kong SAR, China; Institute of Molecular and Cell Biology, Singapore

## Abstract

Amongst all the internal gene segments (PB2. PB1, PA, NP, M and NS), the avian PB1 segment is the only one which was reassorted into the human H2N2 and H3N2 pandemic strains. This suggests that the reassortment of polymerase subunit genes between mammalian and avian influenza viruses might play roles for interspecies transmission. To test this hypothesis, we tested the compatibility between PB2, PB1, PA and NP derived from a H5N1 virus and a mammalian H1N1 virus. All 16 possible combinations of avian-mammalian chimeric viral ribonucleoproteins (vRNPs) were characterized. We showed that recombinant vRNPs with a mammalian PB2 and an avian PB1 had the strongest polymerase activities in human cells at all studied temperature. In addition, viruses with this specific PB2-PB1 combination could grow efficiently in cell cultures, especially at a high incubation temperature. These viruses were potent inducers of proinflammatory cytokines and chemokines in primary human macrophages and pneumocytes. Viruses with this specific PB2-PB1 combination were also found to be more capable to generate adaptive mutations under a new selection pressure. These results suggested that the viral polymerase activity might be relevant for the genesis of influenza viruses of human health concern.

## Introduction

Influenza A viruses with all 16 haemagglutinin (HA) and 9 neuraminidase (NA) subtypes can be isolated from aquatic avian species and this avian viral gene pool is believed to be responsible for the genesis of pandemic viruses [1], [2], [3]. There were three human influenza pandemics in the last century. The pandemic 1918 H1N1 virus was suggested to be of avian origin [4], [5], [6]. By contrast, the 1957 H2N2 and 1968 H3N2 viruses acquired the surface protein(s) (H2 and N2 for H2N2; H3 for H3N2) and polymerase basic 1 (PB1) gene segments from avian strains [7]. Previous studies revealed that the HA and NA surface proteins might play key roles on the zoonotic transmission of influenza virus [8], [9]. Other studies suggested that the host-range specificity of influenza virus is a multigenic trait [10], [11], [12], [13].

The emergence of highly pathogenic (HP) H5N1 avian influenza virus, with its ability to infect humans, has caused serious pandemic concerns [14], [15]. The virus was initially confined to Asia, but it has spread to different continents over the last few years [16], [17], [18]. Thus far, these viruses caused more than 400 confirmed human H5 cases [19] and prompted several mass culling of poultry. The mortality rate of human H5 infections is over 60%. The remarkable disease severity of H5 infections might be partly explained by its ability to provoke exaggerated proinflammatory cytokine and chemokine expressions [20], [21], [22], [23]. Currently, H5 viruses can only be transmitted between humans with a very limited efficiency, indicating that these viruses still do not fully adapt to humans. However, with the experiences learnt from the last 3 pandemics, these H5 viruses or other viral subtypes might gain this ability by introducing adaptive mutations or by reassorting with a human virus.

Growing evidences suggested that the influenza viral trimeric polymerase complex formed by polymerase basic 2 (PB2), polymerase basic 1 (PB1), and polymerase acid (PA) might be associated with viral virulence and/or interspecies transmission [11], [24], [25], [26], [27], [28]. Previous studies revealed that the viral polymerase subunits from human isolates might not be fully compatible to those isolated from avian strains [29]. Others further identified that some residues in these polymerase subunits might control host-restriction [29], [30], [31], [32], [33]. The amino acid position 627 in the PB2 protein was shown to be a critical determinant for viral virulence and host specificity [31], [34], [35], [36]. Interestingly, of all the internal protein gene segments, avian PB1 is the only segment that was reassorted into the human H2N2 and H3N2 viruses. These recurrent events suggested that the acquisition of avian PB1 segment might pose biological advantages to the reassorted viruses. However, the underlying reason for the repeat introductions of avian PB1 into the human viruses is still an enigma. The N- and C-terminal regions of PB1 interact with PA and PB2, respectively, to form a heterotrimeric polymerase complex [37], [38], [39], [40], [41], [42], [43]. In addition, PB1 contains conserved RNA-dependent RNA polymerase motifs, nucleotide binding domains, and viral/complementary RNA (vRNA/cRNA) binding sites [44]. Therefore, it was hypothesized that the reassortments of viral polymerase genes in the pandemic H2 and H3 might modulate the viral polymerase activity, thereby helping the progeny viruses to adapt to humans [45], [46].

In this study, we investigated the compatibility of viral polymerase subunits isolated from a mammalian H1 virus and an avian H5 virus. Specifically, it is of our interest to determine whether recombinant viruses with enhanced viral polymerase activity might have any implications to human infections.

## Materials and Methods

### Cells and viruses

Primary chicken embryonic fibroblasts (CEF) were prepared from specific pathogen free embryonic eggs (Jinan Spafas Poultry Co, Ltd). CEF, 293T human embryonic kidney cells and Madin-Darby canine kidney (MDCK) cells were maintained in minimum essential medium (MEM) supplemented with 10% fetal calf serum (FCS), 1% penicillin, and 1% streptomycin at 37°C. Primary human monocyte-derived macrophages were prepared as described [20]. The differentiated macrophages were cultured in Macrophage Serum Free medium (MSFM, Invitrogen) for one day prior to viral infections. Primary human alveolar epithelial cells (type I pneumocytes) were isolated from patient non-tumor lung tissues as described [23].

All viruses studied in this investigation were synthetically generated by reverse genetic techniques. Indo5 is a clade 2.1 H5N1 virus and does not transmit efficiently in mammals [47].

### Viral infections

Cells infected at multiplicities of infection (MOI) of 0.01 and 2 were used to study virus replication kinetic and cytokine expression profiling, respectively. After one hour of virus absorption, the virus inoculums were washed out and replaced with MEM supplemented with 0.5% FCS (for MDCK), MSFM (for primary macrophage) or small airway growth medium (for pneumocytes; Cambrex Bioscience Walkersville). All viral culture media were supplemented with 0.6 mg/L penicillin, 60 mg/L streptomycin, and 2 mg/L N-p-tosyl-L-phnylalanine chloromethyl ketone (TPCK) treated trypsin (Sigma). The infectivity of the studied viruses were confirmed by staining representative infected cells with anti-NP immunofluorescence antibody (Imagen, oxoid, UK)

### Generation of recombinant influenza viruses

Recombinant virues were generated by reverse genetic techniques [48]. The identities of these viruses or the introduced mutations were confirmed by sequencing. Viral titres were determined by routine plaque assays. Experiments involving infectious recombinant viruses were conducted in biological safety level 3 facilities.

### Expression of recombinant vRNPs

Plasmids expressing WSN PB2, PB1, PA and NP proteins (pcDNA-WSN-PB2, pcDNA-WSN-PB1, pcDNA-WSN-PA, pcDNA-WSN-NP) and WSN NA vRNA (pPolI-NA-RT) were used to generate recombinant RNPs as described [49]. Briefly, 1 µg of each of the plasmids was transfected into 293T cells by Lipofectamine 2000 (Invitrogen) as instructed by the manufacturer. The medium of transfected cells was replaced by MEM with 10% FCS and 1% P/S at 6 hours post-transfection. For the luciferase reporter assay (see below), a model luciferase vRNA expression plasmid (pPolI-Luc-RT) modified from pPolI-NS [49] was used to replace pPolI-NA-RT in the transfection.

For expressing chimeric avian-mammalian vRNPs, the corresponding genes from Indo5 were subcloned into pcDNA-3A (pcDNA-Indo5-PB2 pcDNA-Indo5-PB1, pcDNA-Indo5-PA and pcDNA-Indo5-NP) as described [49].

### Luciferase assay for viral polymerase activities

Various combinations of PB2, PB1, PA and NP protein expression plasmids and the pPolI-Luc-RT were transfected into 293T cells [50]. In addition, a GFP expression plasmid was also co-transfected into the cells and the GFP signals from the transfected cells were used to normalize the data. Initial experiments showed that data with or without the normalization are comparable. For determining the polymerase activities of these recombinant vRNPs in avian cells, the human PolI promoter sequence of pPolI-Luc-RT was replaced by a CEF derived PolI promoter sequence as described [51]. At 48 hours post-infection, treated cells were lysed by Steady-Glo assay reagent (Promega) for 5 minutes and the luminescence was measured by a luminometer (Victor3, PerkinElmer). All data and standard deviations were determined from three independent experiments.

### RNA extraction and cDNA synthesis

Total RNA from transfected or infected cells were harvested by using a RNeasy mini kit (Qiagen). Genomic DNA was digested by DNase Turbo (Ambion) for 30 minutes prior to reverse-transcription reactions. 1.5 µg of total RNA was reverse transcribed by SuperScript II reverse transciptase (Invitrogen) and 10 pmol of vRNA specific primer (5′-AGCAAAAGCAGG-3′) [52] or 50 pmol of oligo-dT primer was used in each reaction.

### Primer extension assay

RNA from MDCK infected at a MOI of 2 was harvested at 6 hours post-infection. Primer extension was performed as described previously [53]. Briefly, 1.5 µg of heat-denatured total RNA was reversed transcribed in a reaction containing 6 pmol of fluorescent primer (5′-Cy3-TGGACTAGTGGGAGCATCAT-3′ for NA vRNA or 5′-Cy5-TCCAGTATGGTTTTGATTTCCG-3′ for NA cRNA/mRNA), 50 U of SuperScript H RNase H- reverse transcriptase (Invitrogen), 1× first-strand buffer (Invitrogen), 1 mM deoxynucleoside triphosphate and 20 mM dithiothreitol. Reactions were incubated at 45°C for 90 minutes. The fluorescent products were resolved in 10% denaturing polyacrylamide gels and the images were analyzed by an imaging analyzer (Typhoon 8600 variable mode imager, Amersham Biosciences). For studying RNA from vRNP-expression cells, transfected cells were harvested at 48 hours post-transfection.

### Cytosol/nucleus fractionation

Nuclear proteins from transfected 293T cells were isolated by incubating PBS washed cells in five times packed cell volume of hypotonic buffer A (10 mM Hepes pH 7.9, 1.5 mM MgCl_2_, 10 mM KCl, 0.5 mM DTT, and complete mini-EDTA-free protease inhibitor cocktail tablet (Roche)) for 10 minutes on ice, followed by passing the lysate through a 27G syringe for multiple times [54]. The lysates were centrifuged at 1,000 g for 5 minutes at 4°C and the pellet was resuspended with buffer S1 (0.25 M sucrose and 10 mM MgCl_2_). The resuspended pellet was overlayed on buffer S2 (0.35 M sucrose and 0.5 mM MgCl_2_), followed by a centrifugation (1,500 g for 5 minutes at 4°C). The pellet was washed with buffer S2, followed by a centrifugation at 1,500 g for 5 minutes at 4°C. The nuclear pellet was stored at −80°C until use.

### Co-Immunoprecipitation

Plasmid pcDNA-PAtap, which expresses a TAP-tagged WSN PA [55], was used to replace pcDNA-WSN-PA to generate TAP-tagged RNPs. Transfected cells were treated with lysis buffer [50 mM HEPEs, pH 8.0, 200 mM NaCl, 25% Glycerol, 0.5% Igepal CA-630 [Sigma], 1 mM β-mercaptoethanol, 0.1 mM PMSF, and complete mini-EDTA-free protease inhibitor cocktail tablet (1 tablet/10 ml; Roche)] at 48 hours post-transfection. The TAP-tagged PA and its interacting proteins were co-purified with immunoglobinlin G-Sepharose (GE Healthcare) and washed with binding buffer [10 mM HEPEs, pH8.0, 150 mM NaCl, 10% Glycerol, 0.1% Igepal CA-630 (Sigma), and 0.1 mM phenylmethylsulfonyl fluoride]. The purified proteins were released to 2× SDS sampling loading buffer by boiling.

### Western blot analysis

Proteins were resolved by 6–12% gradient SDS-polyacrylamide gels and transferred to PVDF membranes (Amersham Biosciences). Proteins were analyzed by Western blotting using anti-Pol II (N-20 and A-10, Santa Cruz Biotechnology), anti-phosphorylated Pol II (8A7, Santa Cruz Biotechnology), anti-β-actin (C4, Santa Cruz Biotechnology), anti-PB2 (PB2/20 and PB2/22) [56], anti-PA [57] primary antibodies and the corresponding HRP-conjugated secondary antibodies. Signals were detected by the ECL Plus detection system as instructed (Amersham Biosciences).

### Quantification of cytokine mRNA by real-time quantitative RT-PCR

Total RNA from infected macrophages and pneumocytes were analyzed by real-time PCR assays as described [20], [23]. Cells were infected at a MOI of 2 and total RNA was harvested at 3, 6 and 8 hours post-infections. The mRNA levels of TNF-α, interferon-β, RANTES (regulated on activation, normal T cell expressed and secreted) and IP-10 (interferon-gamma-inducible protein-10), influenza M1 and beta-actin were determined.

### ELISA assays

The concentrations of TNF-α, IP-10 and RANTES proteins were measured by ELISA assays (R&D Systems, Minneapolis, MN, USA). Samples were first irradiated with ultraviolet light (CL-100 Ultra Violet Cross linker) for 15 min to inactivate any infectious virus before the ELISA assays were done. Cytokine protein levels in the samples and control standards were measured as previously described by us [23]. Previous experiments had confirmed that the dose of ultraviolet light used would not affect cytokine concentrations as measured by ELISA [20].

### Selection of oseltamivir-resistant recombinant viruses

MDCK cells infected at a MOI of 0.1 were treated with oseltamivir at a concentration which can reduce the viral titre by about 99%. Briefly, the wild type and the mutant were passaged in MDCK cells in the presence of 1.52 nM of oseltamivir. In each passage, cells were infected with the passaged viruses (∼MOI of 0.1). At day 2 post-infection, the viral titre in the culture supernatant was determined by plaque assays. Progeny viruses from trial 1 (passage 7) and trial 2 (passage 6) were plaque purified. To test the drug susceptibilities of the purified viruses, MDCK cells infected with 0.1 MOI of these viruses were incubated with 2-fold serially diluted oseltamivir in quadruplicate and the drug concentration required for inhibiting 50% of cytopathic effect (IC_50_) was determined at 48 hours post-infection.

### Statistical analysis

Unless otherwise specified, all data were analyzed on a SPSS platform (SPSS Inc., Chicago). The main and interaction effects of various factors on viral polymerase activities were analyzed by full factorial ANOVA (Univariate General Linear Model) with the log transformed dataset. The statistically significant differences were determined by t-test or Analysis of Variance followed by Bonferroni Post hoc tests (p<0.05). Correlations were determined with bivariate correlations procedures (Pearson's correlation coefficient, p<0.05).

## Results

### Compatibility between PB2, PB1, PA and NP from mammalian and avian strains in human cells

**Figure 1:**
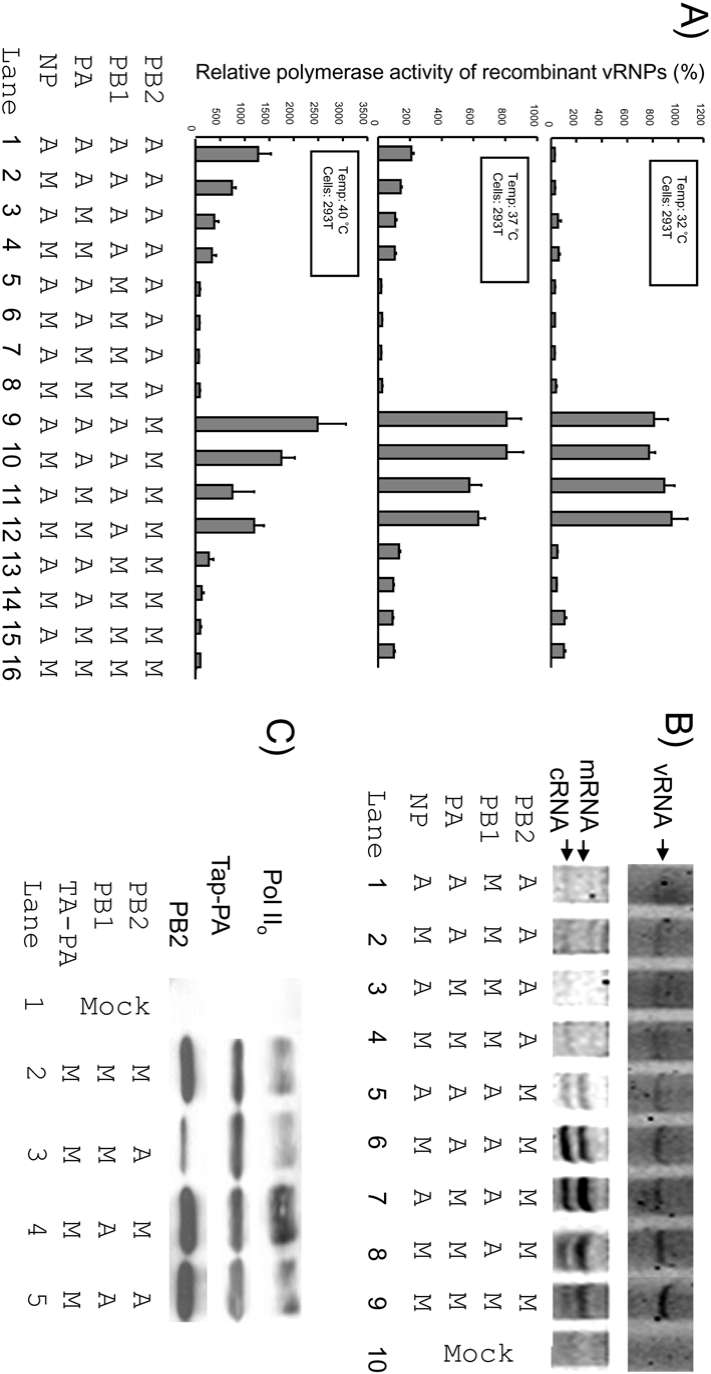
Characterization of recombinant vRNPs generated from transfected cells. The origins of PB2, PB1, PA and NP in each recombinant vRNP were as shown (A = avian, M = mammalian). A) Luciferase reporter assay for influenza viral polymerase activity. Polymerase activities (mean±SE) of recombinant vRNPs in 293T cells incubated at 32°C (top panel), 37°C (middle panel) and 40°C (bottom panel). All data were determined from three independent experiments. The polymerase activities of WSN were set as 100% as references. B) Detection of NA mRNA, cRNA, and vRNA by primer extension assays. Signals for the mRNA, cRNA and vRNA were as shown. C) Immunoprecipitation of TAP-tagged PA. Nuclear lysates expressing different combinations of chimeric viral polymerase complexes were immunoprecipitated by immunoglobinlin G-Sepharose. The amounts of PB2 and Pol II_o_ coimmunoprecipitated with TAP-PA were determined by Western blot techniques.

To study the compatibility between avian and human PB2, PB1, PA and NP at different temperature, we employed a full factorial analytical approach to study these 5 independent factors (i.e. temperature and origin of PB2, PB1, PA and NP). A H1N1 virus (A/WSN/33, WSN) and a H5N1 virus (A/Indonesia/5/05, Indo5) were used as the prototype strains in the study. The WSN PB2 contains a Lys at position 627, whereas Indo5 contains a Glu at the same position. Recombinant vRNPs with all 16 possible combinations were generated in transfected cells and the polymerase activities of these vRNPs were determined by a luciferase reporter assay (Fig. 1A). These mutants were hereafter named according to the source of their genes in the order of PB2, PB1, PA and NP (A: avian; M: mammalian). The readings were then examined by using full factorial univariate Analysis of Variance (ANOVA) so as to determine the relative importance of these factors on influenza viral polymerase activities (Table S1). As shown in Fig. 1A, the origins of PB2 and PB1 were found to have significant effects on viral polymerase activities (F>500; P<0.001). Recombinant vRNPs with either mammalian PB2 or avian PB1 were found to have higher polymerase activities than their counterparts (e.g. compare **M**AAA vs **A**AAA and A**A**AA vs A**M**AA). The results also agreed with previous findings [58], [59] that the incubation temperature has highly significant effects on the viral polymerase activity (F = 271; P<0.001). By contrast, the origins of NP and PA were found to be insignificant and marginally significant (P<0.01; F<7), to the polymerase activity, respectively. This analysis, however, did not exclude the possibility that the origins of these PA and NP are critical for other virological processes.

With these data, we tried to identify whether any of these 2 independent factors might have statistical “interaction” (i.e. synergistic or antagonistic) effects on the viral polymerase activity. Of all 10 possible combinations, 5 pairs (PB2-PB1, PB1-PA, PB2-Temp, PB1-Temp and PA-Temp) were found to be statistically significant (Table S1). Interestingly, a strong PB2-PB1 interaction effect was observed (Table S1, compare the F values amongst these pairs). This demonstrated that the compatibility between PB2 and PB1 is critical to the polymerase activity. Indeed, recombinant viral RNPs with the mammalian PB2-avian PB1 pair (Fig 1A. lanes 9–12) were all found to have strong polymerase activities. By contrast, all vRNPs with the avian PB2-mammalian PB1 pair had reduced polymerase activities (lanes 5–8). These observations were confirmed by testing recombinant vRNPs derived from a clade 1 H5N1 virus (A/VN/1203/03) and the WSN (data not shown). Besides, a relatively weak, but statistically significant, PB1-PA interaction effect was observed. Viral RNPs with an avian PB1-PA pair were found to have stronger polymerase activities than their counterparts in most of the studied conditions. The above findings might be partly explained by the fact that PB1 physically interacts with PB2 and PA for the formation of viral polymerase complex [60].

In addition to the statistical interaction effects between viral polymerase subunits, the temperature-PB2, termperature-PB1 and temperature-PA pairs were found to be statistically significant (Table S1). As shown in Fig. 1A, except the vRNPs with the avian PB2-mammalian PB1 pair (lanes 5–8), all the avian-mammalian chimeric vRNPs had stronger polymerase activities than the WSN control at 40°C. By contrast, except those with the mammalian PB2-avian PB1 pair (Fig. 1A, lane 9–12), all of these recombinant vRNPs were found to be less active than the WSN at 32°C. These agreed with previous findings that human influenza viruses are adapted to replicate in the upper respiratory tract at 33–37°C, whereas avian influenza viruses prefer to replicate in the gut at around 41°C [61], [62]. It should be noted that the vRNPs with the avian PB2-mammalian PB1 pair (Fig. 1A, lanes 5–8) and those with the mammalian PB2-avian PB1 pair (Fig. 1A, lane 9–12) had the weakest and strongest polymerase activities, respectively, at all the studied temperature. These findings reiterated the dominant roles of PB2-PB1 combinations in our experimental setting.

NA-specific primer extension assays were also used to detect NA vRNA, cRNA and mRNA isolated from the transfected cells. Low levels of NA vRNA and cRNA were detected from cells expressing vRNPs with the avian PB2-mammalian PB1 pair (Fig. 1B, lanes 1–4). The mRNA level of these samples was below the detection limit of the assay. By contrast, recombinant vRNPs with the mammalian PB2-avian PB1 pair were found to be very active (Fig. 1B, lanes 5–8). In particular, the cRNA and mRNA generated from these recombinant vRNPs were usually more abundant that the WSN control (Fig. 1B, lane 9).

The above results showed that different combinations of PB2 and PB1 might have differential effects on the polymerase activity. To elucidate the underlying mechanism account for this phenomenon, we selected representative polymerase complexes that have a strong (Fig. 1C, lane 4), moderate (Fig. 1C, lane 5) or weak polymerase activity (Fig. 1C, lane 3) for further characterizations. We first investigated the abundances of these trimeric polymerase complexes formed in the nucleus. Nuclear proteins from cells expressing different combinations of TAP-tagged polymerase complex were immunoprecipitated with immunoglobinlin G-Sepharose. As shown in Fig. 1C, the amounts of TAP-PA of these samples were similar. The PB2 signals from the reactions with the avian PB2-avian PB1 pair (Fig. 1C, lane 5) and with the mammalian PB2-avian PB1 pair (lane 4) were comparable to the WSN control (Fig. 1C, lane 2). However, the PB2 signal from the sample with the avian PB2-mammalian PB1 combination (Fig. 1C, lane 3) was much reduced. These indicated that this specific PB2-PB1 combination, together with PA, might be less capable of forming heterotrimeric polymerase complexes.

Functional viral polymerase complexes were previously shown to bind to the hyperphosphorylated form of RNA Polymerase II (Pol II_o_) [63]. We therefore detected the amount of vRNP-bound Pol II_o_ in the nuclear fraction (Fig 1C, top panel). Interestingly, the Pol II_o_ signal from the reaction with the mammalian PB2-avian PB1 pair (Fig. 1C, lane 4) was much stronger than that of the control (lane 2). By contrast, a reduced Pol II_o_ signal was observed from the reaction with the avian PB2-mammalin PB1 pair (Fig. 1C, lane 3). These results suggested the polymerase complex with the mammalian PB2-avian PB1 combination might be more capable to recruit cellular transcription machinery to initiate viral RNA synthesis.

**Figure 2:**
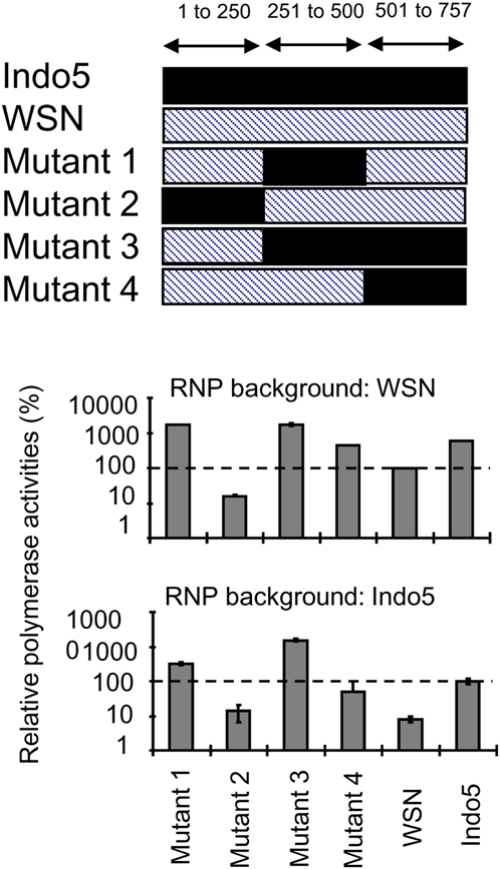
Polymerase activity of vRNPs with chimeric PB1. Various combinations of chimeric PB1 (Mutants 1 to 4, right panel) derived from mammalian (hatched bar) and avian (solid bar) PB1 were tested in the luciferase reporter assay in 293T cells incubated at 37°C. The length of PB1 fragment in each region was indicated. The effect of these chimeric PB1 on vRNP in a WSN (left top panel) or Indo5 (left bottom panel) background were shown. The activities of the wild-type control in the corresponding backgrounds were set as 100% for references.

Some efforts had also been made to map the PB1 and PB2 regions that might modulate the polymerase activity. Chimeric WSN-Indo5 PB1 proteins were generated (Fig. 2) and tested in the luciferase reporter assay. Mutant 2 had a weak polymerase activity in both vRNP backgrounds. As the N-terminal 1/3 of PB1 contains the ORF for PB1-F2 [64], results from the Mutant 2 suggested that the enhanced viral polymerase activity observed in our study is unlikely due to the H5 PB1-F2 protein. The polymerase activities of the Mutant 1, 3 and 4 were similar to or better than the Indo5 wild-type control. Results from the Mutants 1 and 4 further suggested that both the middle 1/3 and C-terminal 1/3 of Indo5 PB1 can stimulate the polymerase activity. Using a similar experimental design, the C-terminal 1/3 of mammalian PB2 was found to responsible for the enhanced viral polymerase activity (Fig. S1). As we primarily interested in studying the implication of influenza polymerase activity on the viral infection, we did not perform systematic mutagenic studies on these genes.

### Compatibility between PB2, PB1, PA and NP from mammalian and avian strains in avian cells

**Figure 3:**
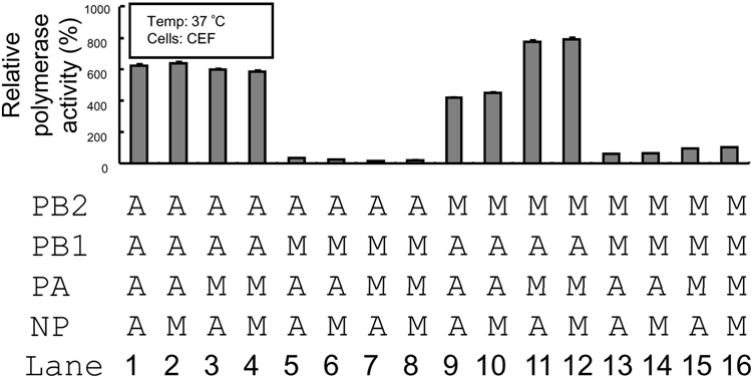
Luciferase reporter assay for influenza viral polymerase activity. Polymerase activities (mean±SE) of recombinant vRNPs generated in CEF cells at 37°C are as shown.

We also tested the compatibility of these polymerase subunits and NP in chicken embryonic fibroblasts (Fig. 3). Using these data and the equivalent data from the human cells (Fig. 1A, middle panel), we performed statistical analysis to study the relative importance of these factors (i.e. origins of PB2, PB1 PA, NP and cells) on the viral polymerase activity. Of all these parameters, the origin of PB1 was shown to have a prominent effect on the polymerase activity (P<0.001) (Table S2). All recombinant vRNPs with the avian PB1 were found to have higher luciferase activities than their counterparts (e.g. compare lanes 9–12 to 13–16 in Fig. 3). Moreover, the origin of cells (P = 0.014) and the origin of PB2 (P = 0.01) was also found to affect the polymerase activity as expected. For example, viral RNPs with an avian PB2 were shown to have much stronger polymerase activities in CEF (Fig. 3, lanes 1–4), but not in human cells (Fig. 1A, lanes 1–4) (P<0.05). These phenomena might be explained by the Lys/Glu polymorphism at position 627 of these PB2 [59]. Because of this cell-type effect, the effect of PB2-PB1 combination on the polymerase activity was not considered to be statistically significant in this dataset (Table S2). Nonetheless, viral RNPs with the mammalian PB2-avian PB1 pair (Fig. 3, lanes 9–12) and with the avian PB2-mammalian PB1 pair (Fig. 3, lanes 5–8) still had the strongest and lowest polymerase activities, respectively, in avian cells. Taken all these together, our data demonstrated that both the origin of PB2 and the origin of PB1 might be critical for viral RNA synthesis in our experimental setting. Specifically, vRNPs with a mammalian PB2 and an avian PB1 were found to have strong viral polymerase activities in all the studied conditions.

### Generation of recombinant viruses with chimeric vRNPs

**Figure 4:**
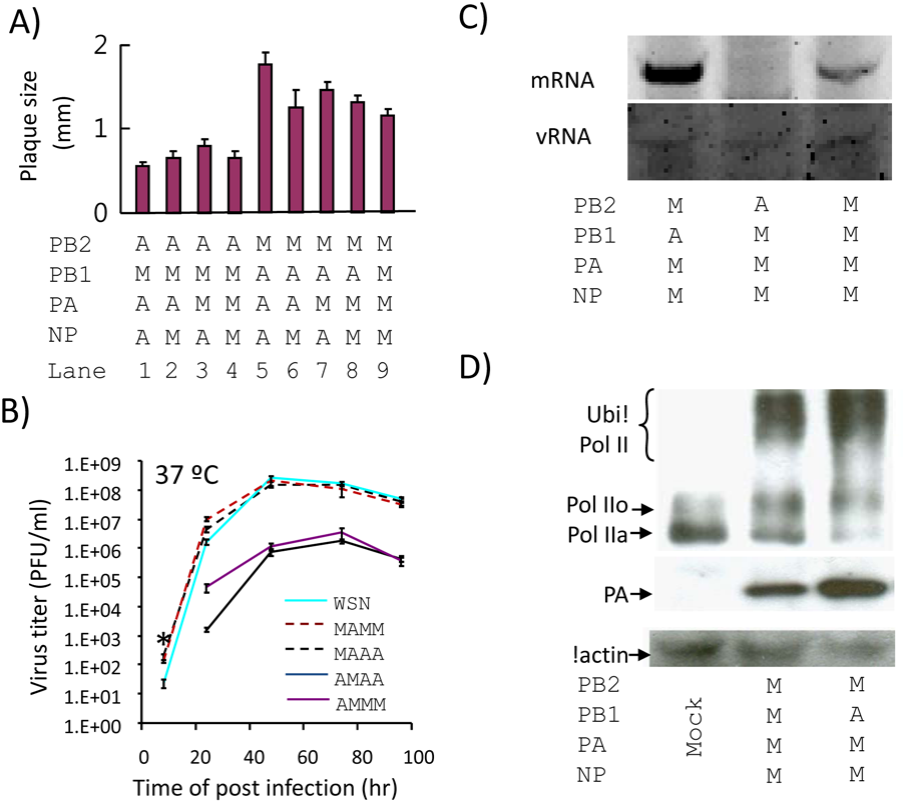
Characterization of recombinant viruses with chimeric polymerase complexes. The origins of PB2, PB1, PA and NP in each recombinant virus was as shown (A = avian, M = mammalian). A) Plaque size (mean±SE) of the wild type (MMMM) and recombinant viruses in MDCK cells at 72 hours post-infection. B) Growth properties of the WSN (MMMM) and recombinant viruses in MDCK cells. The number of infectious progeny viral particles generated from MDCK cells infected with the corresponding virus at a MOI of 0.01 was determined by standard plaque assay. Mutant AMAA and AMMM were significantly attenuated (ANOVA, p<0.05). *At 8 hours post-infection, the amounts of infectious progeny of MAAA and MAMM were significantly higher than the wild type control (t-test, p<0.05). C) NA-specific primer extension assays. Total RNA from MDCK cells infected with influenza virus at a MOI of 2 was harvested at 6 hours post-infection. Signals for the mRNA, cRNA and vRNA were as shown. D) Western blot analysis of influenza PA and cellular Pol II from infected MDCK cells. Total cell lysates from cells infected at 2 MOI were harvested at 6 hours post-infection. Signals for PA and ubiquitinated (Ubi-Pol II), hyperphosphorylated (II_o_) and hypophosphorylated (II_a_) Pol II were indicated. β-actin was used as a control. The PA level of the MAMM mutant was more abundant than the wild type (MMMM), a further illustration of the faster transcription kinetics of this chimeic vRNP. The experiment was repeated three times with comparable results.

To elucidate the possible implications of the above findings to the viral lifecycle, vRNP combinations with the strongest (Fig. 1A, lanes 9–12) and weakest (Fig. 1A, lanes 5–8) polymerase activities were introduced into recombinant viruses by reverse genetic techniques. In addition, to avoid possible influences from the other avian H5 genes (i.e. HA, NP, M and NS), these viruses were all generated in a background of WSN. As shown in Fig. 4A, all recombinant viruses with the avian PB2-mammalian PB1 pair had small plaque phenotypes (t-test, P<0.05) (lanes 1–4). Mutants with the avian PB2-mammalian PB1 pair (AMAA and AMMM) were found to have a 2 log-unit reduction of viral titre when propagated at 37°C (Fig. 4B). By contrast, the plaque sizes of the mutants with the mammalian PB2-avian PB1 combination were all comparable with that of WSN (Fig. 4A). Interestingly, the mammalian PB2-avian PB1 vRNP-containing mutants (MAMM and MAAA) reproducibly had slightly faster replication kinetics than the wild type at the first studied post-infection time point (t-test, P<0.05). The maximum viral titres of these two mutants in the subsequent time points were similar to the wild-type control. But one should not neglect the fact that, at the late stage of viral infection, other factors might be the limiting factors for the virion productions [65], [66], [67], [68].

The growth kinetics of the MAMM mutant at different temperature were also determined (Fig. S2). In all the studied conditions, the viral titres of MAMM mutant were similar or much higher than those of the WSN, demonstrating the MAMM virus could replicate efficiently in a wild range of temperature. In addition, our results also demonstrated that the Indo5 PB1 might play an important role in facilitating the virus replication at high temperature (Fig. S2).

To confirm whether the PB2-PB1 combination would have a direct effect on viral RNA synthesis upon infection, viral RNA generated from representative infected cells was subjected to the NA-specific primer extension assays. As shown in Fig. 4C, viral NA mRNA was highly expressed in MAMM-infected cells, whereas the level of NA mRNA from AMMM infected cells was below the detection limit of the assay. Similarly, the vRNA signal from the AMMM-infected sample was weaker than those of the MAMM sample and WSN control (MMMM). The cRNA signals from these viruses were below the detection limit of the assay.

Apart from binding to cellular Pol II_o_, influenza viral polymerase was shown to reduce the hypophosophorylated Pol II (Pol II_a_) level in infected cells [69]. We therefore characterized the protein expressions of Pol II (Pol II_a_ and Pol II_o_) from infected cell lysates. The levels of Pol II_a_ and Pol II_o_ in infected cells were found to be reduced and enhanced, respectively (Fig. 4D). Interestingly, the Pol II_o_/Pol II_a_ ratio from the MAMM-infected cell was shown to be much higher than that of the wild type. Given the idea that Pol II phosphorylation is a dynamic event [70], our data suggested that the rate of viral RNA synthesis might modulate the phosphorylation status of Pol II. In addition, we consistently observed a characteristic pattern of poly-ubinquinated Pol II in infected cells [71]. As ubiquitination is one of the well known markers for transcriptionally arrested Pol II [72], we believe the modification of Pol II in infected cells might be relevant for viral RNA production [73].

### Induction of pro-inflammatory cytokines and chemokines in primary human cells

**Figure 5:**
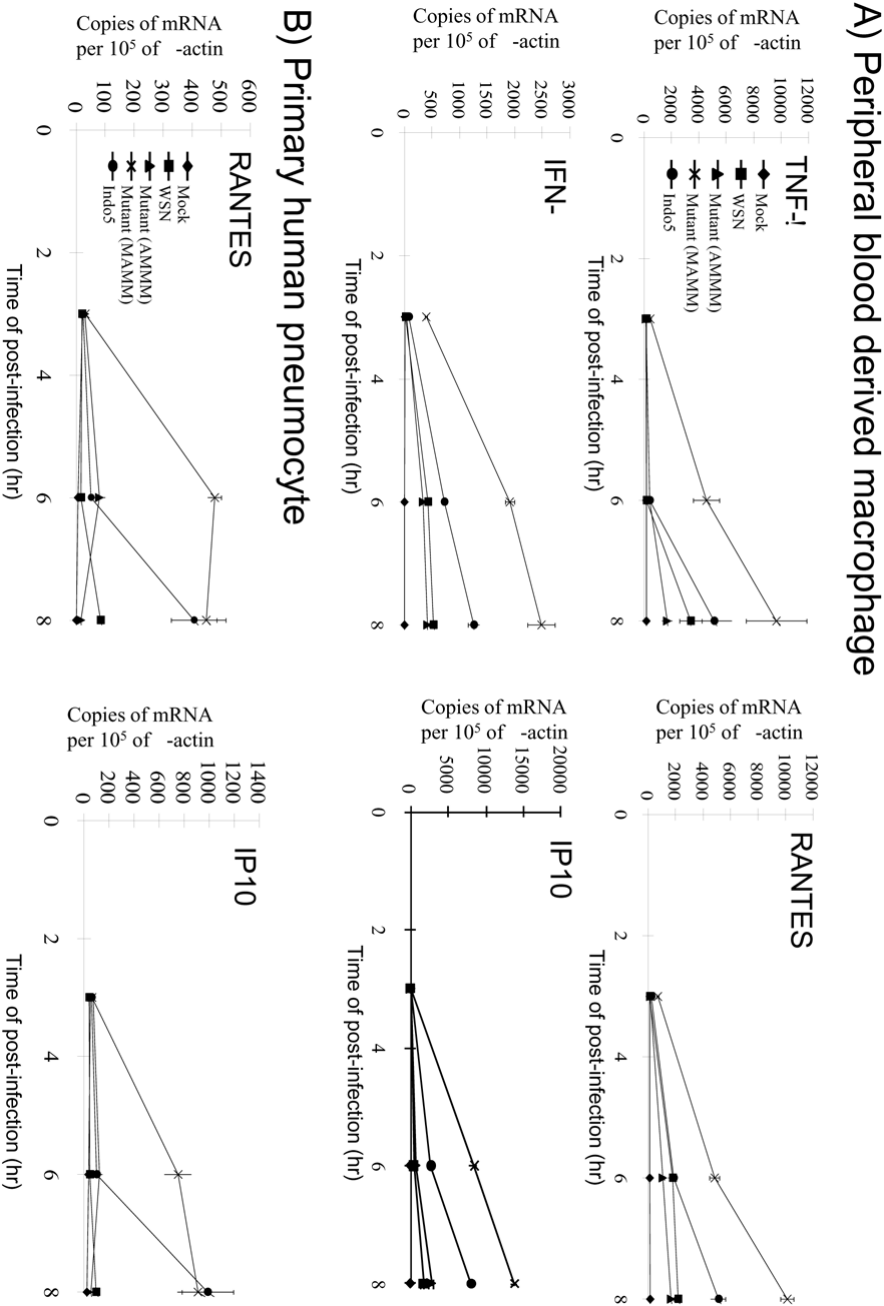
Cytokine and chemokine gene expression profiles (mean±SE) from primary human macrophages (A) and pneuomocytes (B). Total RNA from cells infected at a MOI of 2 was harvested at the indicated time points and tested by the corresponding quantitative RT-PCR assays as indicated. The data were the averages of triplicate assays. The recombinant viruses used in the experiments were as shown.

We and others previously demonstrated that human H5N1 viruses are potent cytokine and chemokine inducers [20], [21], [22], [23]. Subsequent studies further demonstrated that some of the viral proteins from H5N1 viruses might regulate cytokine inductions [20], [74]. To test whether viral polymerase activity might have effects on cytokine/chemokine expressions, representative H1N1 WSN mutants with a weak (AMMM) or a strong (MAMM) polymerase activity were characterized in different primary cell culture models. In addition, a weak (WSN) and a strong (Indo5) cytokine inducer were included as controls. As shown in Fig. 5A, the MAMM mutant was a strong stimulus to primary macrophages and the TNF-α, IFN-β, RANTES and IP-10 gene expressions were found to be higher than those induced by the positive H5N1 control (t-test, p<0.05). By contrast, the AMMM mutant behaved similar to the WSN control. The cytokine/chemokine gene expressions were positive correlated with the amount of viral mRNA produced in the infected cells (Pearson's correlations, p<0.05). Culture supernatants from one of these triplicate experiments were also checked for the TNF-α, IP-10 and RANTES protein concentrations by ELISA and the MAMM-infected cells were found to secrete high levels of these proteins as expected (Table 1).

**Table 1:** Quantitation of TNF-α, IP-10 and RANTES in culture supernatants from infected macrophages collected at different post-infection time points.

Virus	TNF-α (pg/ml)			IP-10 (pg/ml)			RANTES (pg/ml)		
	Time of postinfection			Time of postinfection			Time of postinfection		
	3 hr	6 hr	8 hr	3 hr	6 hr	8 hr	3 hr	6 hr	8 hr
MOCK	U	U	U	U	U	U	1	4	U
WSN	U	U	28	U	U	U	2	11	44
MAMM	U	769	1032	U	75	123	U	100	167
AMMM	U	U	U	U	U	U	4	17	24
Indo5	U	143	391	U	U	128	1	36	106

U: undetectable.

In MAMM-infected primary pneumocytes, similar gene expression patterns were observed, except TNF-α was not detectable as expected [23] (Fig. 5B and data not shown). In summary, these results suggested that the polymerase activity correlate with the cytokine and chemokine inductions.

### Virus with a strong polymerase activity is more ready to generate adaptive mutations under a selection pressure

**Figure 6:**
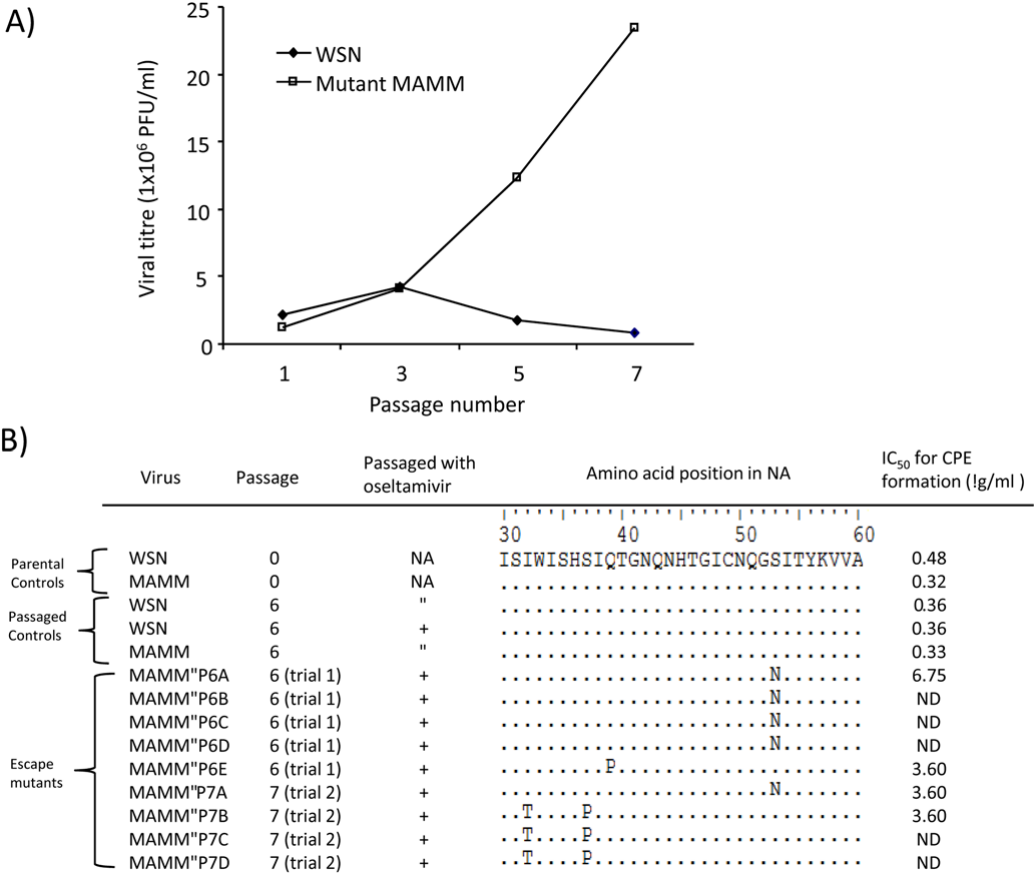
Serial passage of the MAMM mutant in the presence of oseltamivir. A) Viral titers in culture supernatants. The wild type and MAMM mutant were serially passaged in MDCK cells in the presence of oseltamivir. The viral cultures were harvested at 72 hr post-infection and the supernatant from passages 1, 3, 5 and 7 were titrated by standard plaque assays. A representative data set from duplicated experiments was shown. B) Protein sequence of the NA stalk. The passage histories and NA sequences from the parental controls, passaged controls and MAMM escape mutants were as shown. The IC_50_ of oseltamivir towards these viruses were presented.

As the influenza viral polymerase lacks a proofreading activity, the enhanced polymerase activity of the MAMM mutant might allow the virus to generate more mutated viral RNA segments or quasispecies in infected cells. We reasoned that this might enhance the adaptive potential of the mutant. If it was the case, it would be easier for the MAMM to cope with a new selection pressure. To test this hypothesis, we serially passaged the MAMM mutant and the wild-type virus in the presence of a NA inhibitor, oseltamivir. We initially determined the drug concentration that was able to reduce the viral titre by ∼2 log PFU/ml. Under this selected drug concentration, the MAMM virus had a 2 log-unit reduction of viral titre in the early passages (Fig. 6A). Strikingly, the MAMM virus was started to restore its fitness in the subsequent passages (Fig. 6A). From two independent experiments, the viral titres of the MAMM mutant at the late passage were about 15–30 times higher than those found in the early passages. By contrast, the viral titres of the wild-type control from all the studied passages remained low (Fig. 6A). These results suggested that progeny viruses from the MAMM mutant might contain adaptive mutation(s) to counteract the inhibitory effect of the NA inhibitor.

Passaged MAMM mutants from two independent trials were plaque purified (Fig. 6B, N = 9). As oseltamivir is known to induce compensatory mutations in the HA and NA viral segments [75], we therefore sequenced the ORFs of these segments. Apart from a few sporadic mutations in some of these viruses, no consistent mutation or mutational hotspot was identified in the HA or in the globular head of NA. However, all of these “escape” MAMM progeny viruses were found to contain mutations in the NA stalk region (Fig. 6B). Two representative mutants from each trial were selected and tested for their susceptibilities to the drug. As shown in Fig. 6B, the studied escaped mutants had IC_50_ values of oseltamivir about 11 to 21 times higher than those of the controls. These results suggested the potential evolutionary advantages for an influenza virus with a strong polymerase activity.

## Discussion

In this study, we established a statistical method to study the relative importance of different viral/non-viral factors on the viral polymerase activity. In addition, we demonstrated that the potential implications of enhanced viral polymerase activity to human health.

Using WSN and Indo5 as prototype viruses, vRNPs with all 16 possible constellations were tested for their polymerase activities. We primary focused on the factors which had the highest impacts on the polymerase activity. It was demonstrated that both WSN PB2 and Indo5 PB1 could enhance polymerase activity. Besides, the temperature and the origin of the host were found to have a big influence on viral polymerase activities as expected [30], [58], [59]. Our statistical analysis revealed that the PB2-PB1 combination has pronounced effects in our system. Viral polymerases with the WSN PB2-Indo5 PB1 pair were found to have exceptionally high polymerase activities in all the studied conditions. By contrast, Indo5 PB2 and WSN PB1 subunits were found to be highly incompatible. To determine whether a similar PB2-PB1 interaction effect could be detected in other human-avian recombinant vRNPs, we also analyzed data from other similar systematic vRNPs analysis [26], [29], [76]. Our data were in good agreement with the re-interpreted data from two of these previous studies involving recombinant vRNPs derived from human and HP avian strains (H7N1-H1N1 and H5N1-H3N2 pairs) [26], [29]. In these two previous studies, vRNPs with the human PB1-avain PB2 pair were shown to have the highest polymerase activity (Fig. S3). Beside, the introduction of PB1 from a low pathogenic avian H2 virus (A/Mallard/NY/-6750/78) to a human viral polymerase complex was also previously shown to stimulate the viral polymerase activity [26], [29]. Hence, these results indicated that *the PB2-PB1 interaction effect could be found in other recombinant human-avian vRNPs*. On the other hand, Varich et al [77] studied the gene constellations of reassortants between a mammalian H1 and an avian H4. Of 11 isolates that were shown to have a heterologous PB2-PB1 combination in this previous study, 9 of them were found to have a mammalian PB2-avian PB1 gene constellation. Chen et al also studied recombinants derived from an avian H5N1virus and a human H3N2 virus [26]. In this previous report, 62.5% (10 out of 16) of mammalian PB2-avian PB1 recombinants were shown to have efficient virus replications. By contrast, for recombinant viruses with other PB2-PB1 combinations, only 40% of them could replicate in an efficient manner. These results further suggested that vRNPs with the mammalian PB2-avian PB1 combination might be beneficial to some viral strains for robust virus replication.

It should be noted that, however, the PB2-PB1 interaction effect might only applied to a subset of recombinant vRNPs. One should not conclude the findings of this study as a universal phenomenon for all influenza viruses. Other factors, such as the origin of NP or PA, were found to be a critical determinant in controlling the polymerase activity [26], [76]. These results demonstrated that the relative importance of these controlling factors vary between strains. It is worth to mention that all of these previous investigation mainly used the raw data to identify a single viral factor that has the most prominent effect to the polymerase activity [26], [29], [32], [35], [59], [76], [78], [79]. Hence, little is known about the possible interaction effect between these biological or other physical parameters. Using a statistical method, we did not only able to determine the effect of a particular viral polymerase subunit on the viral polymerase activity, but also identified the “interaction effects” that might have a great impact to the vRNP activity. Furthermore, we might use this statistical approach to rank the relative importance of these factors or interaction effects to the viral RNA transcription and replication. It is expected that our new analytical approach might allow systematic studies of different recombinant vRNPs, thereby leading to a better understanding the factors or features that might be critical for efficient viral RNA synthesis.

In this study, MAMM-infected cells were found to have a much reduced Pol II_a_ protein expression. As the Pol II_a_ protein level is negatively correlated with the viral RNA transcription and replication [69], our findings suggested that this specific PB2-PB1 combination is more capable of forming transcriptionally active polymerase complexes. PB1 and PB1-containing protein complexes (i.e. PB2-PB1, PB1-PA and PB2-PB1-PA) were previously shown to bind to various cellular factors [80], [81], [82], [83]. It is possible that the avian PB1 and human PB1 might have different affinities to these proteins, thereby affecting the viral polymerase complex assembly. However, we do not exclude other possibilities which might help to explain this synergistic effect. Further investigations to address these issues are required.

Previous studies demonstrated that the virulence of a HP H5N1 virus might correlate with the polymerase activity [78]. Here, we demonstrated that a H1N1 with a robust vRNP polymerase could induce hypercytokinemia in primary human cells. The cytokine levels induced by this H1N1 mutant were even higher than those from H5-infected cells. Our results indicated that the polymerase activity might be positively correlated to the cytokine gene expressions. It is of interest to study how the MAMM virus can stimulate the cytokine gene expressions. Specifically, it is not known whether the cytokine gene expressions in the MAMM-infected cells were trigged by the enhanced viral polymerase activity or by the increased amount of viral RNA. Influenza viral RNA is known to activate RIG-I and PKR [84]. Further characterizations of these proteins from the MAMM-infected cells might help to better understand this issue. Nonetheless, the high cytokine induction phenotype of the MAMM mutant in human macrophages and pneumocytes is relevant to the pathogenesis of human influenza virus infections.

Using a NA inhibitor to impose a moderate selection pressure to the viruses, the MAMM mutant was found to be more capable of restoring its replication fitness than the wild type. The MAMM escape mutants were confirmed to have an increased resistance to the NA inhibitor and mutations in the NA stalk region. The NA stalk is known to affect the NA activity and viral lifecycle [85], [86]. Introducing point mutations in the NA stalk were previously shown to affect the NA activities [87]. It should be noted that these viruses were intentionally passaged in a suboptimal inhibitory concentration— a condition which allowed both the wild-type and MAMM viruses to yield reasonable viral titers (∼10^6^ PFU/ml). It is possible that compensatory mutations other than those previously described resistance mutations [75] might be generated under this less stringent condition. Further characterizations of these escape mutants are required to elucidate the mechanism responsible for this phenomena. In addition, the fidelity of these recombinant viral polymerase complexes is needed to be fully characterized. Irrespective of the underlying mechanisms account for these observations, our results demonstrated that there is an advantage for influenza viruses to acquire a robust vRNP to cope with a new selection pressure.

The PB1 found in the pandemic human H2 or H3 is of avian origins. It is tempting to speculate that the introduction of avian PB1 in these pandemic reassortants might also enhance the polymerase activities of these viruses, thereby increasing the adaptive potential of these viruses. If this hypothesis was correct, it would be easier for these reassorted viruses to generate adaptive mutations in a new host. Further studies on different viral vRNPs are needed to test this hypothesis.

Taken together, we described the compatibility between avian and mammalian polymerase subunits. Viral polymerase complexes with a WSN PB2 and an Indo5 PB1 were shown to have strong polymerase activities. More importantly, we demonstrated that vRNPs with a strong polymerase activity might be of human health concern. First, this enhanced polymerase activity might induce hypercytokinemia in human cells. Besides, viruses with robust polymerase complexes might be more capable of creating adaptive mutations under a selection pressure. Our findings might add another dimension for identifying influenza viruses of pandemic/zoonotic potential.

## Supporting Information

Table S1 Full factorial analysis of the relative Importance of the incubation temperature and the origin of PB2, PB1, PA, and NP on influenza viral polymerase activity.
(0.37 MB EPS)

Table S2 Full factorial analysis of the relative importance of the origin of PB2, PB1, PA, NP, and cell on influenza viral polymerase activity.
(0.37 MB EPS)

Figure S1 Polymerase activity of vRNPs with chimeric PB2 in 293T cells. Chimeric PB2 (upper panel) derived from the mammalian (open bar) and avian (solid bar) PB2 were tested in the luciferase reporter assay (lower panel) at 37°C. The length of PB2 fragment in each region was indicated. The activities of the wild-type Indo5 vRNP was set as 100%. Chimeric PB2 with its C-terminal 1/3 derived form WSN (iiw, iww or wiw) was able to stimulate the polymerase activity to a level which is comparable to WSN control (www).
(0.55 MB EPS)

Figure S2 Growth properties of the WSN (MMMM) and polymerase mutants at 32°C (A) and 40°C (B). The numbers of infectious progeny viral particles generated from MDCK cells infected at a MOI of 0.01 were determined by standard plaque assays.
(0.44 MB EPS)

Figure S3 Relative polymerase activities of avian-mammalian chimeric vRNPs. Data from Chen et al (A) and Naffakh et al (B) were reorganized and presented in a format used in this study [26], [29]. The origins of PB2, PB1, PA and NP in each recombinant vRNP were as shown (A = avian, M = mammalian). Viral strains used in these studies were shown as indicated.
(0.63 MB EPS)
